# Investigation of continuous effect modifiers in a meta-analysis on higher versus lower PEEP in patients requiring mechanical ventilation - protocol of the ICEM study

**DOI:** 10.1186/2046-4053-3-46

**Published:** 2014-05-20

**Authors:** Benjamin Kasenda, Willi Sauerbrei, Patrick Royston, Matthias Briel

**Affiliations:** 1 Institute for Clinical Epidemiology and Biostatistics, University Hospital Basel, Hebelstrasse 10, 4031 Basel, Switzerland; 2 Department of Medical Biometry and Medical Informatics, University Medical Center Freiburg, Freiburg, Germany; 3 Hub for Trials Methodology Research, MRC Clinical Trials Unit and University College London, London, UK; 4 Department of Clinical Epidemiology and Biostatistics, McMaster University, Hamilton, ON, Canada; 5 Department of Medical Oncology, University Hospital Basel, Basel, Switzerland

**Keywords:** Fractional polynomials, Interaction analysis, MFPI, Continuous predictors, Individual patient data meta-analysis, Acute respiratory distress syndrome

## Abstract

**Background:**

Categorizing an inherently continuous predictor in prognostic analyses raises several critical methodological issues: dependence of the statistical significance on the number and position of the chosen cut-point(s), loss of statistical power, and faulty interpretation of the results if a non-linear association is incorrectly assumed to be linear. This also applies to a therapeutic context where investigators of randomized clinical trials (RCTs) are interested in interactions between treatment assignment and one or more continuous predictors.

**Methods/Design:**

Our goal is to apply the multivariable fractional polynomial interaction (MFPI) approach to investigate interactions between continuous patient baseline variables and the allocated treatment in an individual patient data meta-analysis of three RCTs (N = 2,299) from the intensive care field. For each study, MFPI will provide a continuous treatment effect function. Functions from each of the three studies will be averaged by a novel meta-analysis approach for functions. We will plot treatment effect functions separately for each study and also the averaged function. The averaged function with a related confidence interval will provide a suitable basis to assess whether a continuous patient characteristic modifies the treatment comparison and may be relevant for clinical decision-making. The compared interventions will be a higher or lower positive end-expiratory pressure (PEEP) ventilation strategy in patients requiring mechanical ventilation. The continuous baseline variables body mass index, PaO_2_/FiO_2_, respiratory compliance, and oxygenation index will be the investigated potential effect modifiers. Clinical outcomes for this analysis will be in-hospital mortality, time to death, time to unassisted breathing, and pneumothorax.

**Discussion:**

This project will be the first meta-analysis to combine continuous treatment effect functions derived by the MFPI procedure separately in each of several RCTs. Such an approach requires individual patient data (IPD). They are available from an earlier IPD meta-analysis using different methods for analysis. This new analysis strategy allows assessing whether treatment effects interact with continuous baseline patient characteristics and avoids categorization-based subgroup analyses. These interaction analyses of the present study will be exploratory in nature. However, they may help to foster future research using the MFPI approach to improve interaction analyses of continuous predictors in RCTs and IPD meta-analyses. This study is registered in PROSPERO (CRD42012003129).

## Background

Dichotomizing or categorizing inherently continuous predictor variables raises several issues for statistical analysis and interpretation. These issues include dependence of the statistical significance of the interaction on the number and position of the chosen cut-points, loss of statistical power, and a faulty interpretation of the results if a non-linear association is incorrectly assumed to be linear [1]. To overcome these issues, Royston and Sauerbrei proposed the so-called multivariable fractional polynomials interaction (MFPI) approach to investigate potential treatment modifying effects [2,3]. For continuous variables they propose to estimate a treatment effect function, which avoids the well-known problems caused by categorizing continuous variables. To summarize functions across several studies they suggested a new strategy for meta-analysis [4].

A recent individual patient data meta-analysis of three randomized controlled trials (RCTs) showed that the pre-defined subgroup of patients who suffered from an acute respiratory distress syndrome (ARDS) had a clinical benefit across various endpoints if they were treated with a higher positive end-expiratory pressure (PEEP) ventilation strategy [5,6]. We will use the MFPI approach [2,3] and the new strategy to summarize functions across RCTs [4] to re-analyse this dataset of 2,299 critically ill patients from the previously reported individual patient data (IPD) meta-analysis [5].

### Objectives

The primary aim of the ICEM study is to demonstrate how methodological issues of interaction/subgroup analyses of continuous predictors can be handled by combining a new meta-analysis approach for functions with the MFPI approach. If IPD are available, MFPI allows investigating whether a continuous variable interacts with treatment in one RCT; combination of data from several RCTs strengthens the assessment concerning a treatment modifying effect. When comparing two (or more) treatments in an RCT, several continuous variables (for example, age) are suitable candidates to be investigated as potential modifiers of the treatment effect. The ICEM study will be the first example which combines estimation of treatment effect functions by using MFPI separately in each of several RCTs with a new approach for a meta-analysis of functions. As a secondary aim, we will re-analysis the available IPD data to investigate whether one or more continuous variables have an influence on the comparison of two treatment strategies (higher versus lower PEEP), which is a clinically relevant issue. This paper is an extended version of the registered protocol and shows in an exemplary way how to better use the information from continuous variables if individual patient data from several RCTs are available. In similar projects, it should be obvious how to adapt the relevant steps for a meta-analysis of treatment effect functions.

## Methods/Design

Our protocol is registered on PROSPERO (CRD42012003129 at http://www.crd.york.ac.uk/PROSPERO/display_record.asp?ID=CRD42012003129).

### The dataset

The present interaction analyses will be based on IPD sets from three RCTs identified by a systematic review in 2010 [5,7-9] (Table 1, total of 2,299 patients). These trials investigated the benefits and harms of higher-PEEP ventilation compared to lower-PEEP ventilation in patients with acute lung injury including ARDS. Trial eligibility criteria, literature search strategies, and main results have previously been reported [5]. Standardization of variables and consistency checks have already been performed, thus no more data cleaning will be necessary. Before writing the protocol for the study we have updated the earlier (January 2010) literature search (MEDLINE, EMBASE, CENTRAL) and could not identify additional eligible RCTs. Therefore, the present analysis will focus on the three eligible RCTs from the previous IPD meta-analysis [5].

**Table 1:** Characteristics of ALVEOLI, LOVS, and ExPress studies

	**ALVEOLI (2004)**	**LOVS (2008)**	**ExPress (2008)**
Inclusion criteria	Diagnosis of acute lung injury^a^ with PaO_2_/FIO_2_ ≤ 300	Diagnosis of acute lung injury^a^ and PaO_2_/FIO_2_ ≤ 250	Diagnosis of acute lung injury^a^ with PaO_2_/FIO_2_ ≤ 300
Recruitment period	1999 to 2002	2000 to 2006	2002 to 2005
Number of recruiting hospitals, countries	23, United States	30, Canada, Australia, Saudi Arabia	37, France
Patients randomized to higher versus lower PEEP	276 versus 273	476 versus 509^b^	385 versus 383^c^
Validity:			
Concealed allocation	Yes	Yes	Yes
Follow-up for hospital mortality until day 60	100%	100%	100%
Blinded outcome assessors and data analysts	Yes	Yes	Yes
Early stopping	Stopped for perceived futility	No	Stopped for perceived futility
Experimental intervention	Higher PEEP according to FiO_2_ chart, recruitment maneuvers for first 80 patients	Higher PEEP according to FiO_2_ chart, required plateau pressures ≤ 40 cmH_2_O, recruitment maneuvers	PEEP as high as possible without increasing the maximum inspiratory plateau pressure > 28 to 30 cmH_2_O
Control intervention	Conventional PEEP according to FiO_2_ chart, required plateau pressures ≤ 30 cmH_2_O, no recruitment maneuvers	Conventional PEEP according to FiO_2_ chart, required plateau pressures ≤ 30 cmH_2_O, no recruitment maneuvers	Conventional PEEP (between 5 and 9 cmH_2_O) to meet oxygenation goals
Ventilator procedures	Target tidal volumes of 6 ml/kg of predicted body weight; plateau pressures ≤ 30 cmH_2_O (with exception as above); respiratory rate ≤ 35 breaths/minute, adjusted to achieve arterial pH 7.30 to 7.45; ventilator mode: volume-assist control (except higher PEEP group in LOVS required pressure control); oxygenation goals: PaO_2_ 55 to 80 mmHg and SPO_2_ 88 to 95%; standardized weaning)		

*Abbreviations*: *ALVEOLI* Assessment of Low tidal Volume and Elevated end-expiratory pressure to Obviate Lung Injury, *LOVS* Lung Open Ventilation Study, *ExPress* Expiratory Pressure study, *PaO*_2_ partial pressure of arterial oxygen, *FiO*_2_ fraction of inspired oxygen, *PEEP* positive end-expiratory pressure, *SPO*_2_ oxygen saturation as measured by pulse oximetry.

^a^According to the American-European Consensus Conference [Bernard GR et al., American journal of respiratory and critical care medicine. 1994 Mar;149(3 Pt 1):818-24. PubMed PMID: 7509706].

^b^Includes two patients for whom consent was withdrawn prior to protocol initiation, without patient, family, and caregivers being aware of group assignment (which is 983 patients included in the analysis).

^c^Includes one patient for whom consent was withdrawn prior to protocol initiation, without patient, family, and caregiver awareness of assignment (which is 767 patients included in the analysis).

### Proposed statistical methodology

#### Investigation of interactions

We will use the MFPI approach [2] to investigate the potential treatment (higher versus lower PEEP) modifying effects of each of the continuous variables with respect to a defined outcome. A ‘pair’ of a potential modifier (for example, body mass index (BMI)) and an outcome (for example, in-hospital mortality) will be considered as one investigation. In total, with four potential modifiers and three outcomes we will have twelve investigations. There will be no *P*-value adjustment for multiple investigations. All patients will be analyzed in the group to which they were randomized (intention-to-treat principle). For all analyses, we will use the software STATA version 13.0 (Station College, TX, USA).

**Figure 1:**
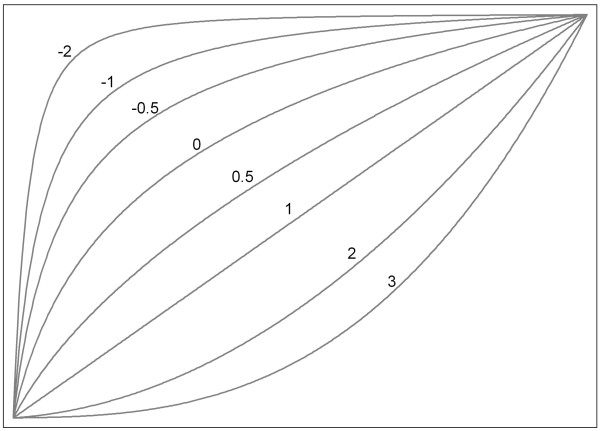
**The variety of curve shapes available with the FP1 family of transformations of a continuous predictor, *x.*** FP1 transformations are simply powers of the form *x*^p^. For example, *x*^p^ with power P = −1 is the reciprocal (1/x) of *x*. These powers are indicated by the numbers on the diagram. Adapted from Royston and Sauerbrei [10].

We will use MFPI with FP2 functions as the most complex allowable function and we will test for an interaction at the 5% level in each trial. FP2 functions are extensions of conventional quadratic functions that provide considerably enhanced flexibility for more realistic modeling in real data. Instead of only powers 1 and 2, they utilize additional combinations of powers of the predictor (see Figure 1 for the powers that may be selected, adapted from Royston and Sauerbrei [10]). Having only two power terms, FP2 functions can exhibit at most one maximum or minimum. We assume that FP2 functions could be a suitable functional form, assuming that patients with extremely high or low values of the continuous predictor might not benefit from the experimental intervention. For each potential effect modifier the functional relationship between this predictor and the outcome will be illustrated using treatment effect functions, irrespective of the *P*-value from the test for interaction. The functional form derived with the MFPI procedure will be checked for potential mismodeling by considering the treatment effect in four subgroups of the predictor of about equal sample size [11]. The analysis strategy needs re-consideration if the estimated treatment effect function disagrees severely with the corresponding results in subgroups, indicating mismodeling of the treatment effect function. For binary outcomes, we will estimate odds ratios with 95% confidence intervals (CIs) to quantify the magnitude of effect. Briel *et al*. had primarily calculated clinically more intuitive relative risks using log-binomial regression instead of odds ratios, but were confronted with computational problems of non-converging log-binomial models in some analyses. For all binary outcomes, they additionally calculated odds ratios and found similar results although event rates for hospital mortality were > 30% in treatment and control groups [6]. Given the similarity of the results we decided to use the logistic regression model in the present study in order to prevent computational issues when applying the MFPI approach. For survival analysis, Kaplan-Meier estimates and hazard ratios with 95% CIs will be presented. Of note, all investigations of a survival outcome will start with a check of the proportional hazards assumption of the effect of treatment in a univariate Cox-model. We will use the Grambsch-Therneau test for this purpose. If the proportional hazards assumption is seriously violated, we will stop the corresponding investigation and will re-consider a suitable strategy for analysis.

#### Individual patient data meta-analysis

Separately for each study we will conduct an MFPI analysis to estimate a treatment effect function. For each modifier-outcome pair, we will use weighted averaging to obtain a summary treatment effect function based on all three studies as previously outlined [4]. We will use a fixed effects approach, because we consider three studies to be too few for a random effect model although a random distribution can be assumed. Usually this averaged treatment effect function is no FP function. It will be plotted to allow for a qualitative assessment of the possible interaction based on the full information of a potential modifier. The individual functions and the averaged function will be the main results to assess whether the variable is a treatment modifier for the specific outcome. We will not conduct any statistical test for the averaged treatment effect function. Combining *P*-values from the individual studies would be one possible way to obtain an overall *P*-value but this is probably not very helpful. More suitable ways to derive an overall *P*-value need to be investigated.

#### Potential clustering of data

We realized that the data of the three independent trials are clustered by recruiting hospitals. Although there is evidence of considerable ‘center effects’ with data from intensive care patients, Briel *et al*. found that the variance among the 90 recruiting hospitals explained very little (0.3%) of the total variance for hospital mortality [6]. Differences in patient baseline characteristics such as age, probability of death in hospital from prognostic scores, and proportion of patients with severe sepsis largely (co-variables in the primary analyses of the present study) explained the between-hospital variance of 2.6% found with a basic hierarchical model including only PEEP group and a categorical trial variable as fixed effects and recruiting hospitals as a random effect. Given the negligible ‘between-hospital’ variance we decided to forgo any consideration of ‘center effects’ in the primary analyses of the present study.

#### Adjustment for confounders

Because of some imbalances regarding age [7,8] and the proportion of patients with severe sepsis [8,9], Briel *et al*. conducted an adjusted analysis for all outcomes [5]. We will adopt this approach, thus each analysis will be conducted with adjustment for the following potential confounders: age (continuous), presence of severe sepsis (yes versus no), and predicted probability of dying in the hospital (based on Acute Physiology and Chronic Health Evaluation II and Simplified Acute Physiology II scores, which have similar accuracy [12,13]). Selection of these potential confounders resulted from a previous Delphi-like structured survey among experts from the intensive care field [6]. We will apply the FP1 function selection strategy to the confounders, with FP1 as the most complex permitted functional form. Including all confounders mentioned above, the model will be determined separately for each of the three outcomes using MFP (1.0, 0.05), independent of treatment. In the notation MFP (alpha 1, alpha 2) the value of alpha 1 gives the significance level for the variable selection part of MFP and alpha 2 the significance level of the function selection procedure for continuous variables [10]. Despite some imbalances in the covariate distributions between PEEP groups mentioned above, univariate approaches will be conducted as sensitivity analyses.

#### Influential points

To circumvent the issue of influential points all continuous variables will be truncated at the 1% and 99%-tile; meaning that any value below the 1%-tile will be replaced by the value of the 1%-tile, and any value above the 99%-tile will be replaced by the value of the 99%-tile. These truncations will be performed for each study separately.

#### Handling missing data

Some of the potential modifiers and variables used for adjustment (see below) have missing values of up to about 10%. In order to use all information in all analyses, we will impute missing values before the main analysis starts. To try to ensure that the missing at random assumption is valid, we will include all outcomes and as many other variables as possible in the imputation models [14]. Five imputations will be created using the multiple imputations by chained equations technique. Only the first imputation will be used in analyses. The remaining four will be reserved for sensitivity analysis of the main findings.

### Description of outcomes and effect modifiers

We selected three clinical important outcomes of interest from a larger list of outcomes used in the analysis by Briel *et al*. [5]:

*In-hospital mortality* at 60 days post randomization (outcome 1a) constitutes the primary efficacy outcome of interest. We will also consider in-hospital mortality as a time-to-event variable (outcome 1b) because we are additionally interested in the timing of mortality events in the randomized groups. Due to the differential follow-up across RCTs beyond day 60 and the fact that the intervention effects happen mainly within the first month, we will censor all surviving patients in the time to event analysis at day 60 as done in the original IPD meta-analysis.

*Time to unassisted breathing (outcome 2)*, which is defined as time from randomization until breathing without mechanical support within the first 28 days is the secondary efficacy outcome of interest. Due to differential follow-up across RCTs for this outcome beyond day 28 and the fact that the intervention effect is supposed to happen before day 28, we will censor patients at day 28 as done in the original IPD meta-analysis. Patients who die before achieving unassisted breathing within the first 28 days will be censored at the day of death. With this procedure we circumvent the competing risk issue in the analysis of this outcome. We are aware of the fact that for prognostic questions, which will not be part of this analysis, cumulative incidence functions would be preferred.

*Pneumothorax requiring chest tube drainage* in first 28 days after randomization (outcome 3, binary variable) is the main safety outcome, because it captures the main potential adverse event directly associated with higher PEEP (experimental intervention). Again, the reason for choosing a 28-day period is that the follow-up for this outcome is different across included trials beyond day 28 and the intervention effect is supposed to happen within the first 28 days. In the present protocol we will not analyze outcome 3 (main safety outcome) because of competing risks with mortality [15]. In the planned clinical report of this work we will refer to the results of the original IPD meta-analysis with respect to outcome 3, because the MFPI methodology has still to be adapted for a competing risk framework. We will deal with competing risks in an addition to this protocol. For the specified efficacy outcomes (outcomes 1a/b and 2) we anticipate no competing risk problems when using cause-specific Cox models.

The following four continuous potential effect modifiers were all pre-specified by Briel *et al*. [5]:

#### Body Mass Index (BMI) at baseline

The BMI is calculated by the ratio of mass in Kg/(Height in m)^2^. There are no data that suggest a certain direction of the treatment effect modification, but Briel *et al*. hypothesized less benefit of higher PEEP in patients with higher BMI [5].

#### Respiratory compliance (RC) at baseline

The RC is estimated by the ratio of:

$$\mathrm{Tidal}\;\mathrm{volume}/\left(\mathrm{Inspiratory}\;\mathrm{plateau}\;\mathrm{pressure}-\mathrm{PEEP}\right)$$

A lower RC would reflect more severe lung injury. Briel *et al*. hypothesized that patients with lower RC have more recruitable lung units and would therefore benefit from higher levels of PEEP. In addition, one could argue that in patients suffering from most severe ARDS, which is commonly associated with very low RC, higher PEEP might no longer provide any benefit.

#### PaO_2_/FiO_2_ ratio at baseline

A low PaO_2_/FiO_2_ reflects impaired blood oxygenation and, therefore, more severe lung injury. Similar to RC, Briel *et al*. hypothesized that patients with a lower PaO_2_/FiO_2_ ratio benefit more from higher PEEP levels. It will be interesting to see how the widely accepted ARDS defining cut-off at 200 mmHg, is reflected in this analysis using the MFPI approach. Using this cut-off, a significant interaction was found by Briel *et al*. [5,6].

#### Oxygenation Index at baseline

The oxygenation index (OI) defined as:

$$\mathrm{mean}\;\mathrm{airway}\;\mathrm{pressure}\;\times \;100/\left[{\mathrm{PaO}}_{2}/{\mathrm{FiO}}_{2}\mathrm{ratio}\right]$$

includes the mean airway pressure and can be regarded as the more reliable marker regarding blood oxygenation compared to the PaO_2_/FiO_2_ ratio alone. The higher the OI, the more severe the lung injury; therefore, Briel *et al*. hypothesized that patients with a higher OI benefit more from higher PEEP levels [5].

Further candidates (for example, age and sex) may be additionally investigated for interaction. Of note, irrespective of the results, all investigations will be included in a summary table similar to the REMARK profile for prognostic studies [16].

## Discussion

The ICEM study is the first example that combines estimation of treatment effect functions by using MFPI with a new approach for a meta-analysis of functions for a clinically relevant issue. The approach requires IPD data, which are available from an earlier meta-analysis project. The present article is an extended version of the registered protocol, and shows in an exemplary way how to better use the information from continuous variables if individual patient data from several RCTs are available. In similar projects, it should be obvious how to adapt the relevant steps for a meta-analysis of treatment effect functions. Besides the new application of the MFPI approach in meta-analysis, the available dataset from three RCTs also offers a unique opportunity to identify potential clinically important interaction effects. All these interaction analyses are exploratory in nature; however, they use the full information for a potential treatment modifier and may help in clinical decision-making. We hope that this project will also foster future research using the MFPI approach to improve interaction analyses of continuous predictors in RCTs and in meta-analyses, provided IPD are available.

## Abbreviations

ARDS: Acute respiratory distress syndrome; BMI: Body mass index; FiO2: Fraction of inhaled oxygen; FP: Fractional polynomial; ICEM: Investigation of continuous effect modifiers; IPD: Individual patient data; MFP: Multivariable fractional polynomial; MFPI: Multivariable fractional polynomial interaction; OI: Oxygenation index; PaO2: Partial arterial pressure of oxygen; PEEP: Positive end-expiratory pressure; RC: Respiratory compliance; RCT: Randomized clinical trial.

## Competing interests

This project has no specific funding. MB is supported by Santésuisse and the Gottfried and Julia Bangerter-Rhyner Foundation. The funding sources have no role in the design and conduct of this study and the writing of this manuscript.

## Authors’ contribution

BK, WS, PR, and MB have designed the study and written the registered protocol and this manuscript. WS and PR developed the MFPI and the meta-analysis approach to combine several functions across studies. MB provided the database. BK, WS, and PR will conduct the analyses. All authors approved the final version before submission.
